# Preclinical anti-arthritic study and pharmacokinetic properties of a potent histone deacetylase inhibitor MPT0G009

**DOI:** 10.1038/cddis.2014.133

**Published:** 2014-04-10

**Authors:** I-N Hsieh, J-P Liou, H-Y Lee, M-J Lai, Y-H Li, C-R Yang

**Affiliations:** 1 School of Pharmacy, College of Medicine, National Taiwan University, Taipei, Taiwan; 2 School of Pharmacy, College of Pharmacy, Taipei Medical University, Taipei, Taiwan

**Keywords:** rheumatoid arthritis, histone deacetylase, epigenetic regulation, synovial fibroblasts, osteoclasts

## Abstract

The pathology of rheumatoid arthritis includes synoviocyte proliferation and inflammatory mediator expression, which may result from dysregulated epigenetic control by histone deacetylase (HDAC). Thus, HDAC inhibitors may be useful for treating inflammatory disease. This was a preclinical study of the HDAC inhibitor, MPT0G009. The IC_50_ values of MPT0G009 for HDAC1, 2, 3, 6 and 8 enzymatic activities were significantly lower than those for the currently marketed HDAC inhibitor suberoylanilide hydroxamic acid (SAHA; vorinostat). In addition, MPT0G009 markedly inhibited cytokine secretion and macrophage colony-stimulating factor/receptor activator of nuclear factor kappa B ligand-induced osteoclastogenesis by macrophages (50 ng/ml each). These MPT0G009 effects on cytokine secretion and osteoclast formation were reduced by the overexpression of HDAC 1 (class I HDAC) and 6 (class II HDAC) in cells, suggesting that these effects were due to the inhibition of its activity. In an *in vivo* rat model, oral administration of MPT0G009 (25 mg/kg) significantly inhibited paw swelling and bone destruction. Furthermore, compared with SAHA, MPT0G009 exhibited longer half-life (9.53 h for oral administration) and higher oral bioavailability (13%) in rats. These results established the preclinical anti-arthritic efficacy and pharmacokinetic parameters of MPT0G009, which may provide a new therapeutic approach for treating inflammatory arthritis.

Rheumatoid arthritis (RA) is a systemic chronic autoimmune disease that results in destructive arthropathy. The complex interactions between the synovial and immune system cells result in synoviocyte proliferation, release of inflammatory cytokines/chemokines that recruit immune cells into the affected joints and activate infiltrated cells, and expression of degradative enzymes, resulting in progressive joint damage.^1^ Thus, these two cell types are key effector cells in RA and provide targets for pathological investigation and drug development.^1, 2^ Classic drugs used for treating RA fall into three categories: nonsteroidal anti-inflammatory drugs (NSAIDs), steroids and disease-modifying anti-rheumatic drugs. However, some adverse effects of these drugs remain major concerns. Recently developed therapies that targeted cytokines have a major impact on the disease course of RA,^3^ however, its usage may be difficult because of increased risks of infection and nonresponse rates.^4, 5^ Therefore, novel treatments that target critical intracellular molecules in synovial inflammation are required.

The imbalance in proinflammatory cytokine networks is central to disease development.^3, 6, 7^ Histone deacetylases (HDACs) are categorized into four categories: class I (HDAC1, 2, 3 and 8); class IIa (HDAC4, 5, 7 and 9) and class IIb (HDAC6 and 10); class III (SIRT1–7); and class IV (HDAC11). These are involved in the post-translational modifications of core histone and nonhistone proteins. Recent proteomic analyses have shown that a substantial number of key signal transduction components and transcription factors that regulate immune responses and inflammation are HDAC substrates.^8, 9^ Thus, HDAC inhibitors have been examined as promising anti-inflammatory agents.^1, 8, 10^ However, there are very few HDAC inhibitors that have been sufficiently developed to undergo clinical trials for RA treatment. One study showed that ITF2357 (givinostat) ameliorated joint inflammation and prevented cartilage and bone destruction in an animal model.^11^ However, a phase II safety and efficacy clinical trial of ITF2357 that evaluated patients with active systemic onset of juvenile idiopathic arthritis, but not those with RA, suggested that HDAC inhibitors still require considerable development for use as RA therapeutics.

We have synthesized 1-arylsulfonyl-5-(*N*-hydroxyacrylamide) indolines derivative HDAC inhibitors. Among these, MPT0G009 (3-[1-(4-methoxybenzenesulfonyl)-2,3-dihydro-1*H*-indol-5-yl]-*N*-hydroxyacrylamide) demonstrated potent cytokine release inhibitory effects.^12^ In this study, we evaluated the anti-arthritic effects of MPT0G009 *in vitro* and in an *in vivo* model and determined the pharmacokinetics and its maximum tolerated dose (MTD). Our results showed that MPT0G009 was >10 times potent than the marketed HDAC inhibitor suberoylanilide hydroxamic acid (SAHA) on HDACs inhibition in the human RA fibroblast-like synoviocytes and rabbit synovial fibroblast cell line, HIG-82. MPT0G009 also had a longer half-life, higher systemic exposure and oral bioavailability than SAHA. Our results show that MPT0G009 is a potential candidate for clinically treating RA.

## Results

### MPT0G009 inhibits HDAC isoform activity

**Figure 1:**
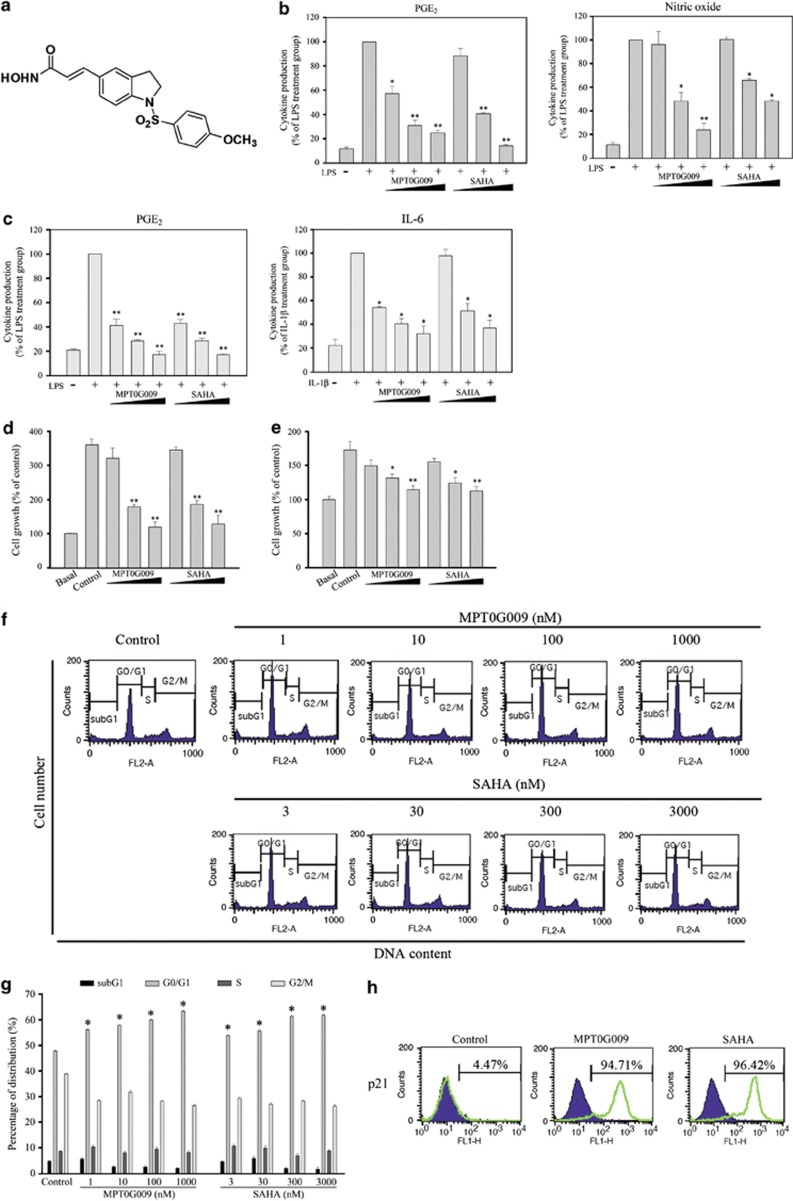
MPT0G009 inhibits inflammatory mediator production and cell proliferation. (**a**) Structure of MPT0G009. (**b**) RAW264.7 cells (1 × 10^6^) and (**c**) RA-FLS (2.5 × 10^4^) were incubated for 30 min with or without MPT0G009 (0.1, 1 or 10 *μ*M) or SAHA (0.3, 3 or 30 *μ*M). Then either LPS (25 ng/ml) was added for 24 h and culture supernatants were assayed for PGE_2_ and nitrites or interleukin (IL)-1*β* (10 ng/ml) was added for 24 h and IL-6 levels were measured. (**d**) HIG-82 synoviocytes and (**e**) RA-FLS (5 × 10^3^) were incubated for 48 h with or without MPT0G009 or SAHA, and their anti-proliferative effects were determined by an SRB assay. (**f** and **g**) RA-FLS (1 × 10^6^) were incubated for 24 h with or without MPT0G009 or SAHA, fixed and then stained with propidium iodide to analyze (**f**) the DNA contents by flow cytometry and (**g**) cell cycle distributions. (**h**) RA-FLS (1 × 10^6^) were incubated for 24 h with or without MPT0G009 (1 *μ*M) or SAHA (3 *μ*M) and then with an anti-p21 antibody to determine the expression of p21 by flow cytometry. Results in (**b**–**e**) and (**g**) are means±S.E.M.'s for three independent experiments, *P<0.05 and **P<0.01 compared with no added inhibitor

The structure of MPT0G009 is shown in Figure 1a. Using kits that contained different recombinant HDAC isoforms, we evaluated the ability of MPT0G009 to inhibit HDAC-mediated deacetylation of lysine residues on the substrates that were provided. As shown in Table 1, MPT0G009 demonstrated potent inhibitory activity for class I HDACs 1, 2, 3 and 8 and for class IIb HDAC6 but not for class IIa HDAC4, with IC_50_ values of 4.62, 5.16, 1.91, 22.48, 8.43 and >10^4^ nM, respectively. The HDAC isoform inhibitory activity of MPT0G009 was clearly greater than that of SAHA, which was used as the reference compound.

### Anti-inflammatory and anti-proliferative effects of MPT0G009

To evaluate the anti-inflammatory effects of MPT0G009, supernatants from cultures of RAW264.7 cells (Figure 1b) and RA fibroblast-like synoviocyte (RA-FLS; Figure 1c) were incubated with different concentrations of MPT0G009 (0, 0.1, 1 or 10 *μ*M) or SAHA (0, 0.3, 3 or 30 *μ*M) for 30 min before and during incubation for 24 h with lipopolysaccharide (LPS, 25 ng/ml) or IL-1*β* (10 ng/ml). These supernatants were then assayed for prostaglandin E2 (PGE_2_), NO and IL-6.

MPT0G009 and SAHA inhibited PGE_2_ production by both cell types, NO production by RAW264.7 cells and IL-6 production by RA-FLS in a concentration-dependent manner; MPT0G009 was more effective than SAHA. As synoviocyte proliferation has a pivotal role in RA pathogenesis, we assessed the effects of MPT0G009 and SAHA at the above mentioned concentrations on the proliferation of HIG-82 synoviocytes (Figure 1d) or RA-FLS (Figure 1e) after 24 or 48 h of incubation (Supplementary Figures 2a and b). These results showed that both inhibitors had similar concentration-dependent anti-proliferative effects on both cell types.

To investigate the effects of MPT0G009 and SAHA on cell cycle progression, cellular DNA contents were determined by flow cytometry. As shown in Figures 1f and g, treating RA-FLS with MPT0G009 (1–1000 nM) or SAHA (3–3000 nM) for 24 h did not increase the subG1 peak, suggesting that these agents did not cause cellular apoptosis. However, G0/G1 phase arrest was observed after treating these cells with all concentrations of both agents. We then examined whether this was attributable to an effect on cyclin-dependent kinase inhibitors, such as p21, by incubating RA-FLS with 1 *μ*M MPT0G009 or 3 *μ*M SAHA for 24 h and assessed the expression of p21 by flow cytometry (Figure 1h) or western blot (Supplementary Figure 2c). We found that treating these cells with either agent significantly increased the expression of p21, which was consistent with their effects on cell cycle distributions.

### MPT0G009 increases histone H3 acetylation in HIG-82 synovial fibroblasts and RA fibroblast-like synoviocytes

**Figure 2:**
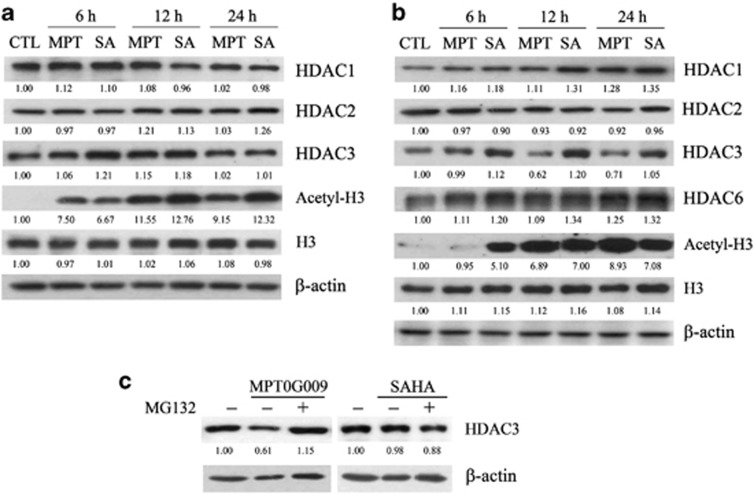
MPT0G009 increases the expression of acetyl histone 3 proteins in synovial fibroblasts. (**a**) HIG-82 cells and (**b**) RA-FLS (1 × 10^6^) in six-well plates were either untreated for 24 h or were incubated for the indicated times with MPT0G009 (3 *μ*M for HIG-82 cells; 1 *μ*M for RA-FLS) or SAHA (60 *μ*M for HIG-82 cells; 30 *μ*M for RA-FLS). Then, cells were harvested and cell lysates were prepared for western blot analysis of the indicated proteins. (**c**) RA-FLSs were incubated for 1 h with or without the proteasome inhibitor, MG132 (1 *μ*M). Cells were then incubated with or without MPT0G009 (1 *μ*M) or SAHA (30 *μ*M) for another 24 h in the continued presence or absence of the inhibitor, after which cell lysates were prepared for western blot analysis of the histone deacetylase inhibitor (HDAC3). Results shown are representative of three independent experiments. The numbers below each blot are the mean quantitative results as measured by densitometry relative to that without MPT0G009 or SAHA

As histone H3 is a target of HDACs, we examined whether a MPT0G009- or SAHA-induced decrease in HDAC activity resulted in changes in histone acetylation in HIG-82 synoviocytes and RA-FLS. Western blots of lysates of HIG-82 synoviocytes that were treated with 3 *μ*M MPT0G009 (Figure 2a) or RA-FLS that was treated with 1 *μ*M MPT0G009 (Figure 2b) for 6, 12 or 24 h showed that there was significant hyperacetylation of histone H3 (Acetyl-H3) starting at 6 h (HIG-82 cells) or 12 h (RA-FLS), and it was maintained for at least until 24 h as compared with those of an untreated control. SAHA treatment of both cell types (60 *μ*M for HIG-82 synovial fibroblasts and 30 *μ*M for RA-FLS) had a similar effect. In addition, treating RA-FLS cells, but not HIG-82 cells, with MPT0G009 for 12 or 24 h resulted in decreased levels of HDAC3 but not of the other isoforms, whereas SAHA had no effect (Figure 2b). This was a proteasome-dependent effect because it was reduced by pretreatment with the proteasome inhibitor, MG132 (1 *μ*M; Figure 2c).

### MPT0G009 inhibits macrophage colony-stimulating factor/receptor activator of nuclear factor kappa-B (NF-*κ*B) ligand (M-CSF/RANKL)-induced osteoclast formation

**Figure 3:**
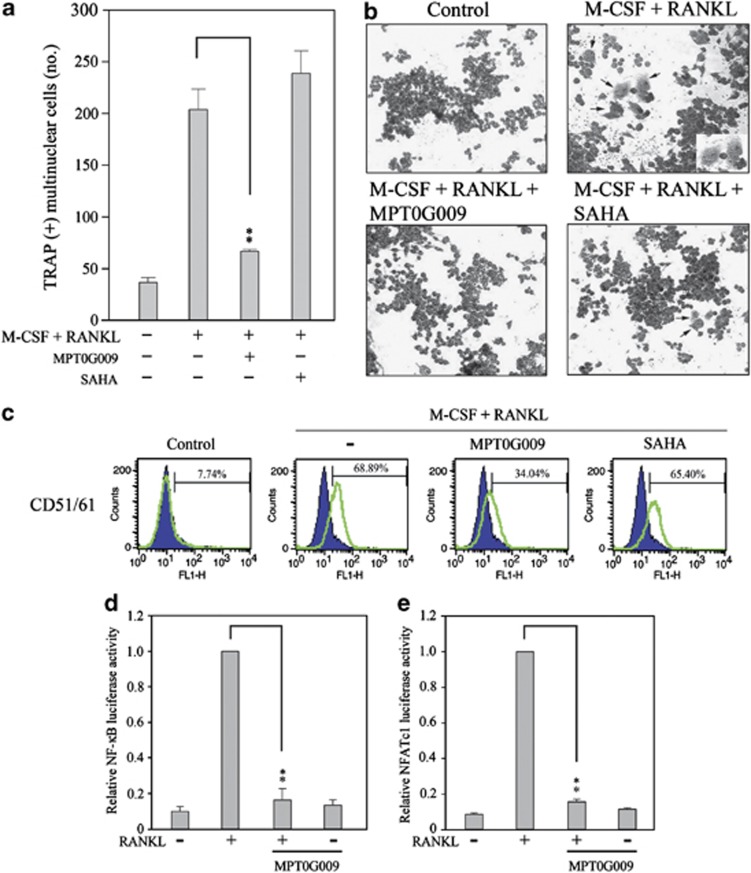
MPT0G009 inhibits the formation of osteoclast-like multi-nuclear cells by RAW264.7 macrophages. (**a**) RAW264.7 cells (1 × 10^3^) were incubated for 30 min with MPT0G009 (5 nM) or SAHA (50 nM), after which M-CSF and RANKL (50 ng/ml each) were added, and incubation was continued for 5 days. Cells were then TRAP stained, and the numbers of TRAP-positive multinuclear cells were counted. (**b**) RAW264.7 cells were incubated with 5 nM MPT0G009 or 50 nM SAHA and treated as in (**a**), after which their morphology was examined by light microscopy. Images are at × 100 magnification, and the arrows indicate differentiated osteoclasts. (**c**) RAW264.7 cells were treated as in (**b**), then incubated with a FITC-conjugated anti-CD51/61 antibody and analyzed by flow cytometry. (**d** and **e**) RAW264.7 cells (1 × 10^5^) were transfected with 1 *μ*g of pGL4.32[luc2P/NF-*κ*B-RE/Hygro] (**d**) or pGL4.30[luc2P/NFAT-RE/Hygro] (**e**) for 24 h and then incubated for 30 min with or without MPT0G009 (5 nM). Then, RANKL (50 ng/ml) was added and incubation was continued for an additional 24 h, after which luciferase activity was determined. Results in (**a**, **d** and **e**) are the means±S.E.M.'s for three independent experiments. **P<0.01 compared with the indicated controls

Bone destruction is one characteristic of RA pathogenesis, resulting in joint dysfunction. Differentiation of mouse macrophages osteoclast-like cells can be induced in the presence of M-CSF and RANKL, which has been used as a model to investigate osteoclast differentiation.^13^ To evaluate the effect of MPT0G009 on osteoclast formation, RAW264.7 macrophages were incubated for 30 min with or without 5 nM MPT0G009 or 50 nM SAHA (Figures 3a and b) before and during treatment with M-CSF/RANKL (50 ng/ml) for 5 days. Subsequently, multinucleate tartrate-resistant acid phosphatase (TRAP)-positive cells were counted. In the absence of an inhibitor, M-CSF/RANKL treatment induced the formation of 205±10 TRAP-positive multinuclear osteoclast-like cells (Figures 3a and b), and 68.9±3.7% of these cells were positive for the osteoclast-specific marker CD51/61 (Figure 3c). The concentrations of MPT0G009 that were used (5 nM) significantly inhibited the formation of M-CSF/RANKL-induced TRAP-positive cells (Figure 3a), and significantly inhibited the expression of the osteoclast-specific marker (Figure 3c). In contrast, SAHA treatment had no effect, even at 50 nM (Figures 3a–c).

We also assessed the effect of MTP0G009 on the DNA-binding activity of NF-*κ*B and NFATc1, two pivotal transcriptional factors involved in RANKL-induced pathways for promoting osteoclast differentiation.^14, 15^ When RAW264.7 cells that had been transiently transfected with reporter plasmids were treated with 5 nM MPT0G009 for 30 min before and during 24-h stimulation with RANKL, MPT0G009 inhibited RANKL-induced NF-*κ*B (Figure 3d) and NFATc1 (Figure 3e) luciferase activity. These results showed that MPT0G009 could inhibit M-CSF/RANKL-induced osteoclast differentiation and signals.

### MPT0G009's inhibitory effects on cytokine production and osteoclast differentiation are reduced by the overexpression of HDAC1 and HDAC6

**Figure 4:**
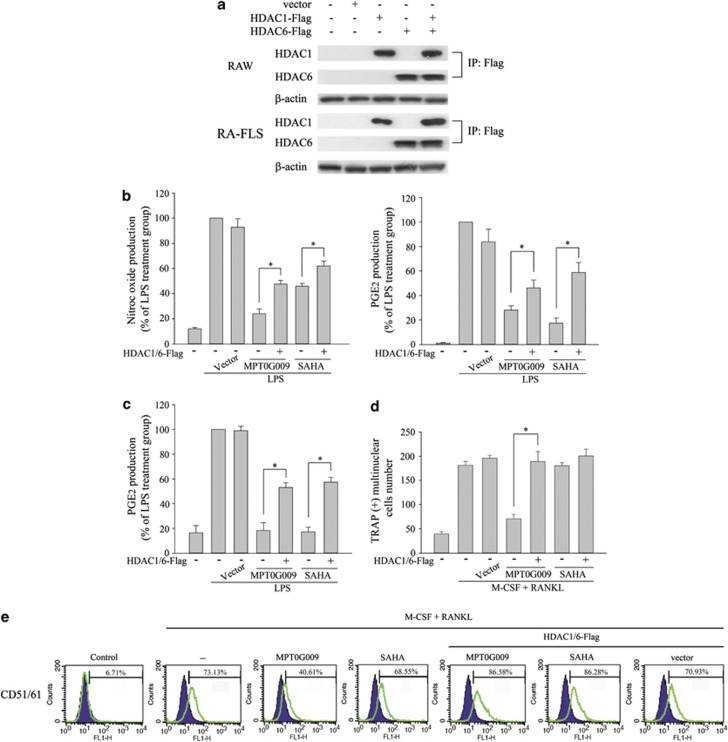
Overexpression of HDAC1 and HDAC6 reduces MPT0G009 inhibition of cytokine secretion and osteoclast differentiation. (**a**) RAW264.7 macrophages or RA-FLS (1 × 10^6^) were transfected for 24 h with 1 *μ*g of an empty vector or a vector encoding for HDAC1-Flag and/or HDAC6-Flag, after which cell lysates were immunoprecipitated with 1 *μ*g of an anti-Flag antibody and immunoblotted for the indicated proteins. (**b**) RAW264.7 cells (1 × 10^6^) and (**c**) RA-FLS (2.5 × 10^4^) that were transfected with an empty vector or vectors encoding for both HDAC1 and HDAC6 as in (**a**) were incubated for 30 min with or without 10 *μ*M of MPT0G009 or 30 *μ*M of SAHA. Then, LPS (25 ng/ml) was added for another 24 h, and culture supernatants were assayed for nitric oxide or PGE_2_. (**d** and **e**) RAW264.7 cells (1 × 10^3^) transfected as in (**b**) were incubated for 30 min with or without 5 nM of MPT0G009 or 50 nM of SAHA, after which M-CSF and RANKL (50 ng/ml each) were added and incubated for 5 days. Cells were then TRAP stained, and the numbers of TRAP-positive multinuclear cells were counted (**d**) or were incubated with a FITC-conjugated anti-CD51/61 antibody and analyzed by flow cytometry (**e**). Results in (**b**–**d**) are means±S.E.M.s for three independent experiments. *P<0.05

Next, we examined whether MPT0G009 inhibited cytokine release and osteoclast formation by inhibiting HDAC activity. As shown in Figure 4a, RAW264.7 macrophages and RA-FLS that were transfected with HDAC1- and/or HDAC6-encoding plasmid(s) expressed the expected isoforms(s). Empty vector-transfected or HDAC1- and HDAC6-coexpressing RAW264.7 macrophages (Figure 4b) or RA-FLS (Figure 4c) were incubated for 30 min with or without 10 *μ*M MPT0G009 or 30 *μ*M SAHA. Then, LPS (25 ng/ml) was added for 24 h, and nitrite and PGE_2_ levels were measured in culture supernatants. These results showed that overexpression of HDAC significantly reduced the inhibitory effects of MPT0G009 or SAHA on LPS-mediated NO or PGE_2_ release (Figures 4b and c) and that of MPT0G009 on M-CSF/RANKL-induced osteoclast differentiation (Figure 4d) as well as the expression of the osteoclast-specific marker, CD51/61 (Figure 4e). Thus, MPT0G009 apparently inhibited cytokine release and osteoclast differentiation by inhibiting HDAC activity.

### MPT0G009 inhibits the development of arthritis in an adjuvant-induced arthritis (AIA) model

**Figure 5:**
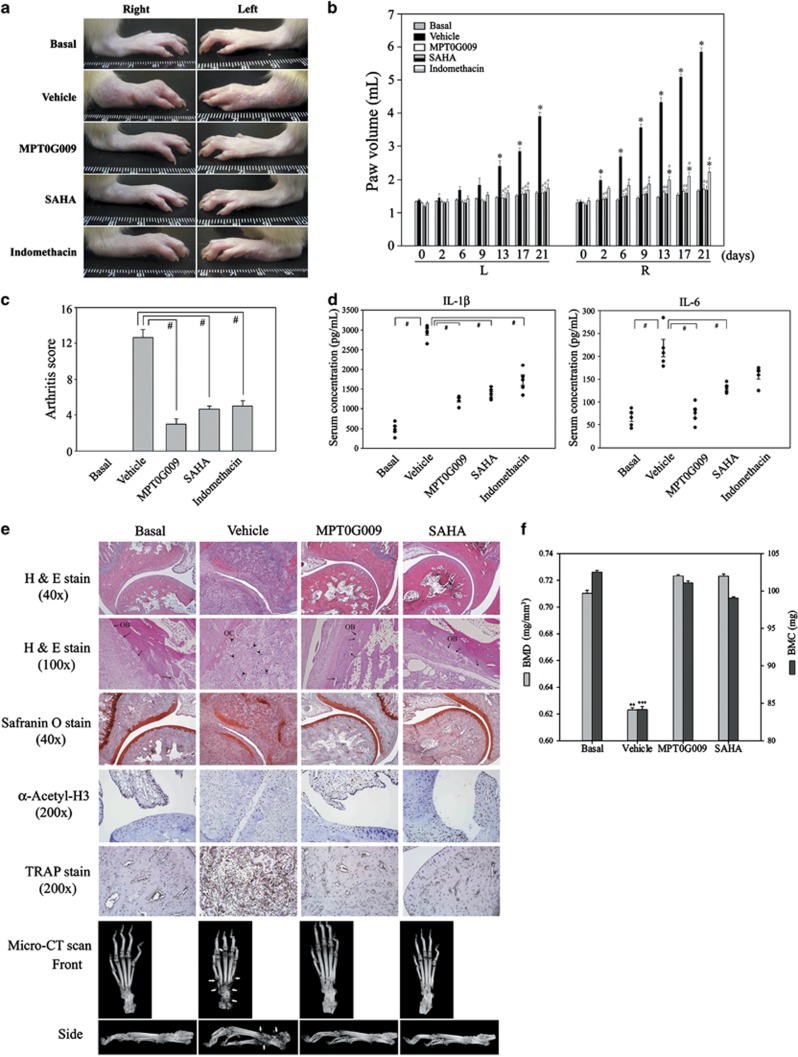
MPT0G009 inhibits the development of arthritis in an AIA model. (**a**) After the onset of arthritis (as described in the Materials and methods section), rats were orally treated with either the vehicle (MPT0G009; 25 mg/kg), SAHA (200 mg/kg) or the positive control indomethacin (1 mg/kg) from days 2 to 21 (19 days). Subsequently, swelling of both hind paws was photographed. (**b**) Hind paw volumes in the indicated group of rats were measured using a digital plethysmometer on the indicated day after AIA induction. (**c**) Arthritis scores on day 21. (**d**) Serum levels of interleukin (IL)-1*β* (left panel) and IL-6 (right panel) on day 21 as measured by enzyme-linked immunosorbent assay (ELISA). (**e**) Top five rows: photomicrographs of ankle joint sections from the different groups stained with hematoxylin and eosin, safranin O, immunohistochemically stained with an anti-acetyl H3 antibody or TRAP stain. Arrows indicate osteoblasts (OB); arrowheads indicate osteoclasts (OC). Bottom panels: micro-CT results (arrows indicate bone erosion). (**f**) The BMD (in mg/mm^3^) and the BMC (in mg) values for the bone tissue of the tarsus were analyzed using CT Analysis Software. Results in (**b**–**d** and **f**) are the means±S.E.M.'s for five independent experiments. *P<0.05 compared with the corresponding day 0 value (**b**); ^#^*P*<0.05 compared with the vehicle-treated control (**b**–**d**); **P<0.01 and ***P<0.001 compared with basal groups (**f**)

Next, we evaluated the *in vivo* anti-arthritic effects of MPT0G009 in a rat AIA model. As shown in Figure 5, compared with the vehicle-treated group, the group treated with 25 mg/kg of MPT0G009 daily from days 2 to 21 had significant reductions in paw swelling (Figure 5a), paw volume (Figure 5b) and arthritis scores (Figure 5c). Similar results were found with SAHA (200 mg/kg) and NSAIDs (indomethacin; 1 mg/kg). MPT0G009 treatment resulted in significant decreases in the serum levels of IL-1*β* and IL-6, as did SAHA and indomethacin treatments (Figure 5d). Furthermore, as shown in Figure 5e, safranin O staining of rat ankle joints showed that MPT0G009 treatment markedly reduced cartilage degradation, and hematoxylin and eosin staining showed that MPT0G009 treatment significantly reduced leukocyte infiltration, synovitis and apparently ameliorated the decrease of osteoblasts. Immunohistochemical staining with an anti-acetyl-histone H3 antibody showed that the MPT0G009-treated group had increased levels of acetyl-histone H3, and TRAP stain demonstrated that MPT0G009 treatment significantly decreased the formation of osteoclasts. The inhibition of synoviocytes proliferation and inflammation by MPT0G009 treatment was also observed (Supplementary Figure 1). In addition, micro-computed tomography (micro-CT) scans showed that MPT0G009 treatment ameliorated bone destruction (Figure 5e) and prevented the decrease of bone mineral density (BMD) and bone mineral content (BMC) (Figure 5f). In these experiments, 25 mg/kg of MPT0G009 had effects similar to 200 mg/kg of SAHA and showed that it had very potent anti-arthritic effects *in vivo*.

### *In vivo* rat pharmacokinetic profiling and MTD of MPT0G009

The pharmokinetic parameters of MPT0G009 in rats after single-dose intravenous (i.v.) and oral administration are summarized in Table 2. After i.v. administration, the half-life of MPT0G009 was 6.74 h, and systemic exposure and clearance were 665 ng h/ml and 5.12 l/h/kg, respectively. After oral administration, MPT0G009 showed *T*_max_=3.43 h, *T*_1/2_=9.53 h and bioavailability (*F*)=13.0% (Table 2). Table 3 shows the MTD data for MPT0G009 in CD-1 mice with a daily × 7 schedule. No significant adverse effects were observed within 3 weeks in a study of mice when MPT0G009 was administrated at a dosage of up to 1000 mg/kg/day.

## Discussion

In this study, we evaluated the preclinical anti-arthritic effects of an orally available HDAC inhibitor, MPT0G009, both *in vitro* and in an *in vivo* model. MPT0G009 was found to inhibit cytokine release by LPS-stimulated macrophages, inhibit synoviocyte proliferation, reduce osteoclast differentiation and ameliorate arthritis progression. In addition, we found that the overexpression of HDAC reduced the inhibitory effects of MPT0G009, showing that its effects were primarily due to a decrease in HDAC activity. Furthermore, we determined the pharmacokinetic parameters and MTD of MPT0G009.

HDAC inhibitors were originally developed as anticancer agents that functioned through multiple mechanisms, such as inducing cancer cell apoptosis and cell cycle arrest.^9, 16^ Recent proteomics analyses have shown that a substantial number of key signal transduction components and transcription factors that regulate immune responses and inflammation are HDAC substrates.^9, 10^ Consistent with these findings, HDAC inhibitors reduce LPS-induced or IL-1-induced expression levels of inflammatory mediators^17, 18^ and inflammatory mediator-induced synovial inflammation and subsequent cartilage destruction.^19^ A majority of these are pan-HDAC inhibitors and target the following two primary classes of HDAC enzymes: class I and class II. Although which HDACs are involved in the processes associated with RA inflammation remains unclear, it has been suggested that several isoforms may have important roles in RA pathogenesis. High HDAC1 levels were observed in RA synovial tissues; these levels correlated with the higher expression of tumor necrosis factor.^20^ HDAC3 knockdown inhibited the expression of VCAM-1 in a human endothelial cell line, thereby reducing monocyte adhesion.^21^ HDAC3 knockdown also inhibited osteoclast differentiation by mouse bone marrow and RAW264.7 cells.^22^ An HDAC6 inhibitor promoted T-regulatory-dependent suppressive activity in an autoinflammatory model.^23^ HDAC8 was upregulated during the later stages of osteoclast development after RANKL treatment.^24^ Therefore, pan-HDAC inhibitors probably exert their therapeutic effects in multiple ways.^9^ In this study, MPT0G009 significantly inhibited the activity of all the four class I HDACs (HDAC1, 2, 3 and 8) and a class IIb HDAC (HDAC6). Thus, it was not unexpected that it suppressed RA development through multiple mechanisms by inhibiting RA-FLS proliferation, osteoclast differentiation and inflammatory mediator release.

We also found that MPT0G009 treatment resulted in proteasome-dependent downregulation of HDAC3. Interestingly, SAHA treatment was found to decrease the levels of HDAC2 and HDAC4 in the cortex and brain stem in the R6/2 Huntington's mouse model.^25^ However, the question of why MPT0G009 induces proteasome-dependent downregulation of HDAC3 cannot be explained. We speculate that it may be because of the inhibition of HDAC6 activity, as HDAC6 regulates *α*-tubulin acetylation and facilitates transportation of degradable proteins from the aggresome to the lysosome.^26, 27^ In addition, an HDAC6 inhibitor resulted in *α*-tubulin hyperacetylation, disruption of the interaction between HDAC6 and dynein, and an increase in the delivery of ubiquitinated proteins to the proteasome.^28^ Our results showed that in RA-FLS, MPT0G009 inhibited HDAC6 activity and also reduced the levels of HDAC3 by proteasomal-mediated degradation. This latter effect was inhibited by the 26S proteasome inhibitor MG-132. However, why MPT0G009 treatment only downregulated the expression of HDAC3 and not that of other HDAC isoforms will require further investigation.

Bone erosion is associated with RA severity and poor functional outcomes.^7^ A recent study showed that a combination of class I and II HDAC inhibitors synergistically inhibited osteoclast activity, and this combination was more effective than a class I or class II inhibitor alone,^24^ suggesting that a pan-HDAC inhibitor may be more effective in suppressing the osteoclast activity. Our results showed that MPT0G009 significantly inhibited RANKL-induced osteoclast formation at a concentration as low as 5 nM, whereas SAHA had little effect even at a concentration as high as 50 nM. We suggest that this may be because MPT0G009 has a greater inhibitory effect than SAHA on both class I and II HDACs. In addition, downregulation of MPT0G009-induced HDAC3 may contribute to its inhibitory effect on osteoclast differentiation because HDAC3 downregulation inhibits osteoclast differentiation in mouse bone marrow and RAW264.7 cells.^22^ Another study showed that SAHA could suppress osteoclastogenesis *in vitro* but at a concentration (300 nM) that is higher than the maximum concentration used in our study (50 nM).^29^

A previous study established the pharmacokinetic parameters for SAHA in rats,^30^ as shown in Table 2. Compared with SAHA, MPT0G009 had a longer half-life and greater systemic exposure after i.v. and oral administration in rats. Furthermore, the absolute oral bioavailability of MPT0G009 was found to be 13.0%, which was better than that of SAHA (6.9%). These pharmacokinetic parameters suggest that MPT0G009 may offer better therapeutic effects when orally administered. In addition, we found no significant adverse reactions with MPT0G009, suggesting that it was well tolerated and safe within the dosage range of up to 1000 mg/kg (Table 3).

Taken together, the superior potency and pharmacokinetic properties of MPT0G009 compared with those of SAHA in this preclinical setting makes it a candidate for clinically treating RA.

## Materials and Methods

### Materials

MPT0G009 and SAHA were synthesized by Dr. Jing-Ping Liou to >98% purity.^12^ The non-conjugated primary antibodies used were against HDAC6 or *β*-actin (Epitomics Inc., Burlingame, CA, USA), HDAC1, HDAC2, HDAC3 or histone H3 (Cell Signaling Technology, Danvers, MA, USA), or acetyl-histone H3 or p21/WAF1/Cip1 (Millipore, Temecula, CA, USA). Fluorescein isothiocyanate (FITC)-conjugated anti-CD51/61 antibodies was from BD Biosciences (San Jose, CA, USA). The labeled secondary antibodies were horseradish peroxidase- or FITC-conjugated anti-mouse or anti-rabbit IgG antibodies (Jackson ImmunoResearch Inc., West Grove, PA, USA). Fluorogenic HDAC1, 2, 3, 4, 6 or 8 assay kits were from BPS Bioscience Corp. (San Diego, CA, USA), colorimetric HDAC activity kits from BioVision Inc. (Milpitas, CA, USA), PGE_2_ immunoassay kits from R&D Systems (Minneapolis, MN, USA) and IL-6 ELISA kits from eBioscience Inc. (San Diego, CA, USA). The pGL4.32[*luc2P/*NF-*κ*B-RE*/*Hygro] and pGL4.30[*luc2P/*NFAT-RE*/*Hygro] plasmids were from Promega Corp. (Madison, HI, USA) and the HDAC1-Flag (plasmid 13820) and HDAC6-Flag (plasmid 13823) plasmids from Addgene Inc. (Cambridge, MA, USA). TurboFect transfection reagent was from Fermentas (Burlington, ON, Canada). Unless otherwise stated, all other chemicals were from Sigma-Aldrich (St. Louis, MO, USA).

### Cell culture

The HIG-82 rabbit synovial fibroblast and RAW264.7 mouse macrophage cell lines were purchased from the Bioresource Collection and Research Center (Hsinchu, Taiwan) and the cells cultured at 37 °C in 5% CO_2_/95% air in, respectively, 90% Ham's F-12 or Dulbecco's modified Eagle medium, both containing 10% heat-inactivated fetal bovine serum (FBS) (Invitrogen Life Technologies, Carlsbad, CA, USA) and 1% penicillin/streptomycin (Biological Industries, Kibbutz Beit Haemek, Israel). Human RA-FLSs from Cell Application Inc. (San Diego, CA, USA) were grown in synoviocyte growth medium from the same supplier.

### Inhibition of recombinant HDAC enzyme activity

Fluorogenic HDAC assay kits were used to assess the ability of HDAC inhibitors to inhibit deacetylation of lysine residues on the substrate by recombinant HDAC1, 2, 3, 4, 6 or 8 according to the manufacturer's instructions.

### HDAC activity assay of cell lysates

Colorimetric HDAC activity kits were used to assay HDAC activity in cell lysates according to the manufacturer's instructions.

### Cell proliferation assay

HIG-82 synoviocytes or RA-FLSs were incubated for 48 h with the indicated concentrations of MPT0G009 or SAHA, then were fixed with 10% trichloroacetic acid, stained for 30 min with sulforhodamine B (SRB; 0.4% in 1% acetic acid), and washed repeatedly with 1% acetic acid, then protein-bound dye was dissolved in 10 mM Tris base solution and the optical density at 510 nm measured.

### Osteoclast differentiation assay

Osteoclast formation was measured by quantifying cells stained for the osteoclast marker TRAP using an acid phosphatase kit (387-A; Sigma-Aldrich). Briefly, the specimens were fixed for 30 s in fixative solution (25.5% citrate solution, 66.3% acetone, 8.2% of 37% formaldehyde), then incubated for 1 h at 37 °C with a mixture of the provided naphthol AS-BI phosphate and tartrate solutions, followed by counterstaining with hematoxylin solution. Osteoclasts were counted as TRAP-positive multinuclear (>3 nuclei) cells using light microscopy. The morphological features of osteoclasts were also photographed.

### Flow cytometry

RAW264.7 cells and RA-FLSs were harvested and washed twice with FACS washing buffer (1% FBS in PBS), followed by incubation at 4 °C for 30 min with antibodies. After three washes with FACS washing buffer, the fluorescence of the cells was analyzed on a FACScan flow cytometer (BD Biosciences). To detect cell cycle progression, cells were incubated with or without the indicated agent for 24 h, washed twice with ice-cold PBS, collected by centrifugation and fixed in 70% (v/v) ethanol for 2 h at −20 °C. They were then incubated for 30 min at room temperature with 0.2 ml of DNA extraction buffer (0.2 M Na_2_HPO_4_ and 0.1 M citric acid buffer, pH 7.8), resuspended in 1 ml of propidium iodide staining buffer (0.1% Triton X-100, 100 *μ*g/ml of RNase A and 80 *μ*g/ml of propidium iodide in PBS), incubated at 37 °C for 30 min in the dark, sorted by FACScan, and analyzed using CellQuest software (BD Biosciences).

### Transfection and reporter gene assay

RAW264.7 cells or RA-FLSs were seeded 1 day before transfection. The plasmids pGL4.32[*luc2P/*NF-*κ*B-RE*/*Hygro], pGL4.30[*luc2P/*NFAT-RE*/*Hygro], HDAC1-Flag, and HDAC6-Flag (1 *μ*g of each) and 1 *μ*l of TurboFect transfection reagent were mixed for 20 min at room temperature, then were added to the cells and the mixtures incubated for 24 h at 37 °C in a humidified atmosphere of 5% CO_2_ in air. For the reporter gene assay, 100 *μ*l of reporter lysis buffer (Promega Corp.) was added, then the cells were scraped off the dishes, centrifuged at 17 000 g for 30 s at 4 °C and the supernatants collected. Aliquots of cell lysates (20 *μ*l) were placed in the wells of an opaque black 96-well microtiter plate, then 40 *μ*l of luciferase substrate (Promega Corp.) was added and the luminescence immediately measured in a microplate luminometer (Beckman Coulter, Krefeld, Germany).

### Pharmacokinetic evaluation

A 1–5 mg/ml dosing solution was preparing by dissolving appropriate amount compound in a mixture of PEG400/DMSO (80 : 20, v/v) for i.v. administration and 1% carboxylmethyl cellulose, 0.5% Tween 80 for oral dosing, respectively. Eight to 10-week-old male Sprague–Dawley rats were obtained from BioLASCO Corp. (Ilan, Taiwan), and tested compound was separately administered to group of rats (*n*=3 per group) i.v. (2 mg/kg dose) by a bolus injection to jugular vein or periorally (20 mg/kg dose). Blood sample (0.15 ml) from 0 h (before dosing) to 24 h was collected from each animal via the jugular vein cannula and stored in ice. Plasma was separated from the blood and centrifuged at 14 000 g for 30 min at 4 °C. All samples were analyzed for the test compound by LC-MS/MS (ABI4000; Applied Biosystems, Foster City, CA, USA). Data were acquired via multiple reactions monitoring. Plasma concentration data were analyzed with standard noncompartmental method.

### Determination of the MTD

The MTD was determined using CD-1 mice at 8–10 weeks of age by dose ranging from 250 to 1000 mg/kg orally once a day for 1 week and then monitored for another 2 weeks. Each dose was tested on six mice in individual independent experiments. MTD was defined as the highest dose that could be given resulting in no drug-related moribund state or death, while temporary body weight loss was within 20%.

### *In vivo* AIA model

Five-week-old male Lewis rats were obtained from the National Laboratory Animal Center (Taipei, Taiwan). Complete Freund's adjuvant (CFA) was prepared by suspending heat-killed *Mycobacterium butyricum* (Difco, Sparks, MD, USA) in mineral oil at 3 mg/ml. CFA-induced arthritis was induced by intradermal injection of 100 *μ*l of the CFA emulsion into the base of the right hind paw on day 0. MPT0G009 (25 mg/kg), SAHA (200 mg/kg) or vehicle was given daily by gavage from days 2 to 21. On days 0, 2, 6, 9, 13, 17 and 21, the animals were weighed and both hind paw volumes measured using a digital plethysmometer (Diagnostic & Research Instruments Co. Ltd, Taipei, Taiwan). On day 21, micro-CT of the paws was performed by the Core Facilities Center of the National Research Program for Biopharmaceuticals using an *in vivo* micro-CT scanner (Skyscan 1176, Bruker Corp., Kontich, Belgium) at 18 *μ*m resolution and 180° scanning with a rotation step of 0.8° per image, 300 ms integration time, 70 keV photon energy and 350 *μ*A current. The reconstructed volumes from micro-CT scans were analyzed by CT Analysis Software (CTAn, Bruker Corp.). Quantification of volumetric BMD and bone volume (BV) was performed in a defined bone area ranging 12 mm from tarsals to the end of the calcaneus. The BMC was described by the product of BV and BMD. On day 21, the rats were killed and specimens from the phalanges and ankle joints stained with hematoxylin–eosin or safranin O for histopathological assessment by the National Laboratory Animal Center. Intensity of arthritis was scored as described previously from 0 to 4.^19^ All four legs were scored, so the highest possible arthritic score was 16.

### Ethics

Animal experiments were approved by the Institutional Animal Care and Use Committee of the National Taiwan University College of Medicine (IACUC number: 20110376 and 20110303).

### Data analysis

The data are expressed as the mean±S.E.M. and were analyzed using one-way ANOVA. When ANOVA showed significant differences between groups, Tukey's *post hoc* test was used to determine the pairs of groups showing statistically significant differences. A *P-*value of <0.05 was considered statistically significant.

## Figures and Tables

**Table 1:** IC_50_ values for MPT0G009 or SAHA for different recombinant HDAC isoforms

	**IC**_**50**_ **(nM) of HDAC isoforms enzyme**^a^					
**Compound**	**HDAC1**	**HDAC2**	**HDAC3**	**DHAC8**	**HDAC4**	**HDAC6**
MPT0G009	4.62±0.81	5.16±0.76	1.91±0.22	22.48±2.16	>10^4^	8.43±0.72
SAHA	45.97±5.62	96.90±8.43	33.87±2.38	542.83±32.73	>10^4^	15.50±1.38

a Data represent the mean±S.E.M. from three replicate experiments

**Table 2:** Intravenous and oral pharmacokinetic parameters of MPT0G009 and SAHA in rats

**Parameters**	**MPT0G009**		**SAHA**^**30**^	
	**i.v. 2 mg/kg**	**p.o. 20 mg/kg**	**i.v. 2 mg/kg**	**p.o.5 mg/kg**
CL (l/h/kg)	5.12	—	8.04	—
*V*_*d*_ (l/kg)	25.21	—	3.92	—
*T*_*1/2*_ (h)	6.74	9.53	0.64	1.13
AUC_(0-inf)_ (ng** **h/ml)	665	1265	278	49.40
*T*_max_ (h)	—	3.43	—	0.17
*C*_max_ (ng/ml)	—	221	—	83.90
*F* (%)	—	13.0	—	6.9

**Table 3:** The MTD of MPT0G009

**Compound**	**Dose (mg/kg)**	**Schedule**	**Mouse no.**	**Change of body weight (%)**			**Lethality (%)**
				**Day 7**	**Day 14**	**Day 21**	
MPT0G009	250	p.o., qd × 7	6	+8.7±0.5	+17.0±0.4	+22.6±0.6	0
	500	p.o., qd × 7	6	+1.6±0.3	+16.5±0.6	+24.0±0.9	0
	1000	p.o., qd × 7	6	+4.4±0.6	+12.0±0.7	+15.3±0.8	0

Abbreviation: MTD, maximum tolerated dose

Data in the column of ‘change of body weight' represent mean±S.E.M.

## Abbreviations

RA: rheumatoid arthritis
HDAC: histone deacetylase
NF-*κ*B: nuclear factor kappa B
PCR: polymerase chain reaction
RANKL: receptor activator of nuclear factor kappa-B ligand
M-CSF: macrophage colony-stimulating factor
