# Supplementary figures and images for: Preclinical anti-arthritic study and pharmacokinetic properties of a potent histone deacetylase inhibitor MPT0G009
Source: Cell Death Dis. 2014 Apr 10;5(4):e1166–. doi: 10.1038/cddis.2014.133 (PMC5424110; doi:10.1038/cddis.2014.133)

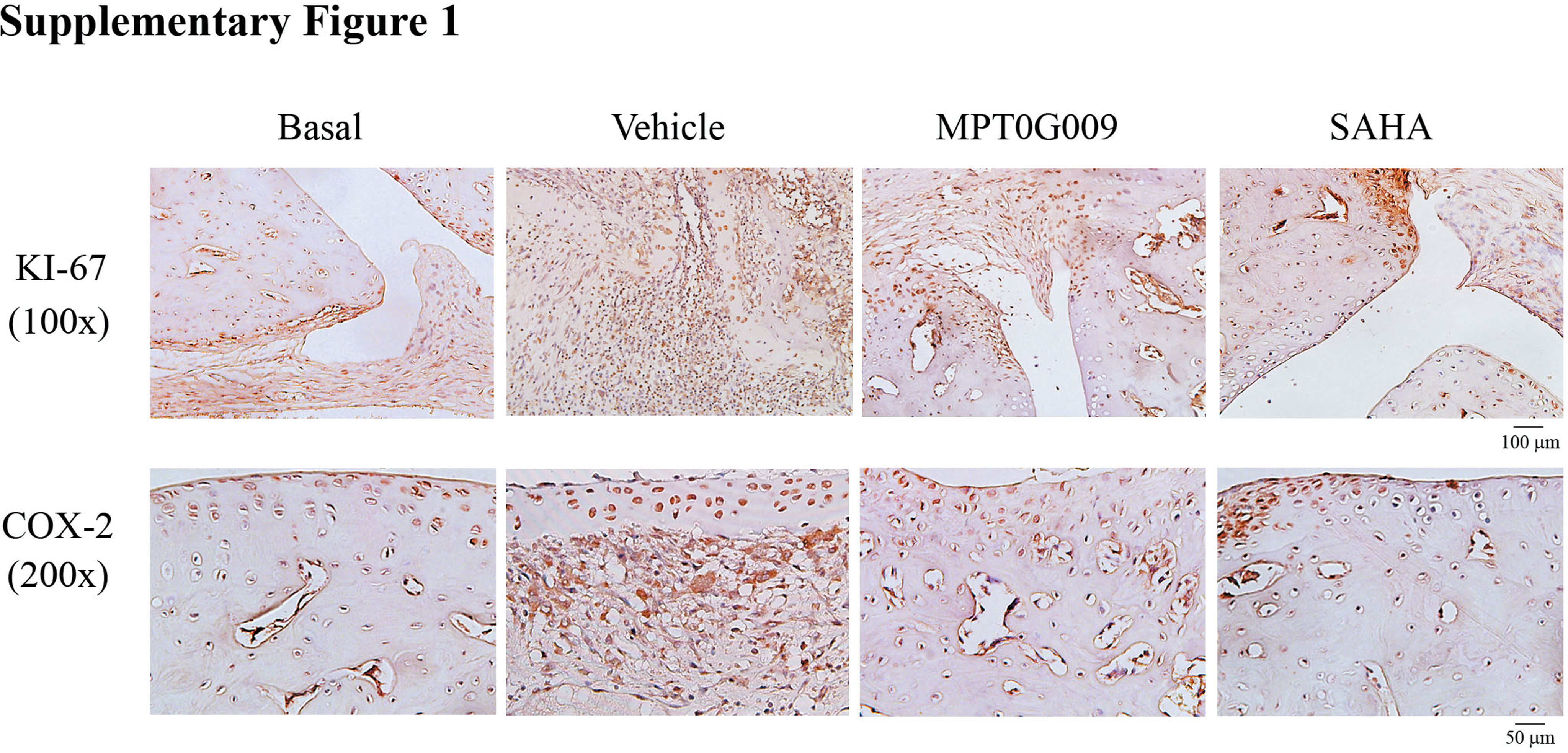

Supplement: Supplementary Figure 1 [file cddis2014133x1.tif]

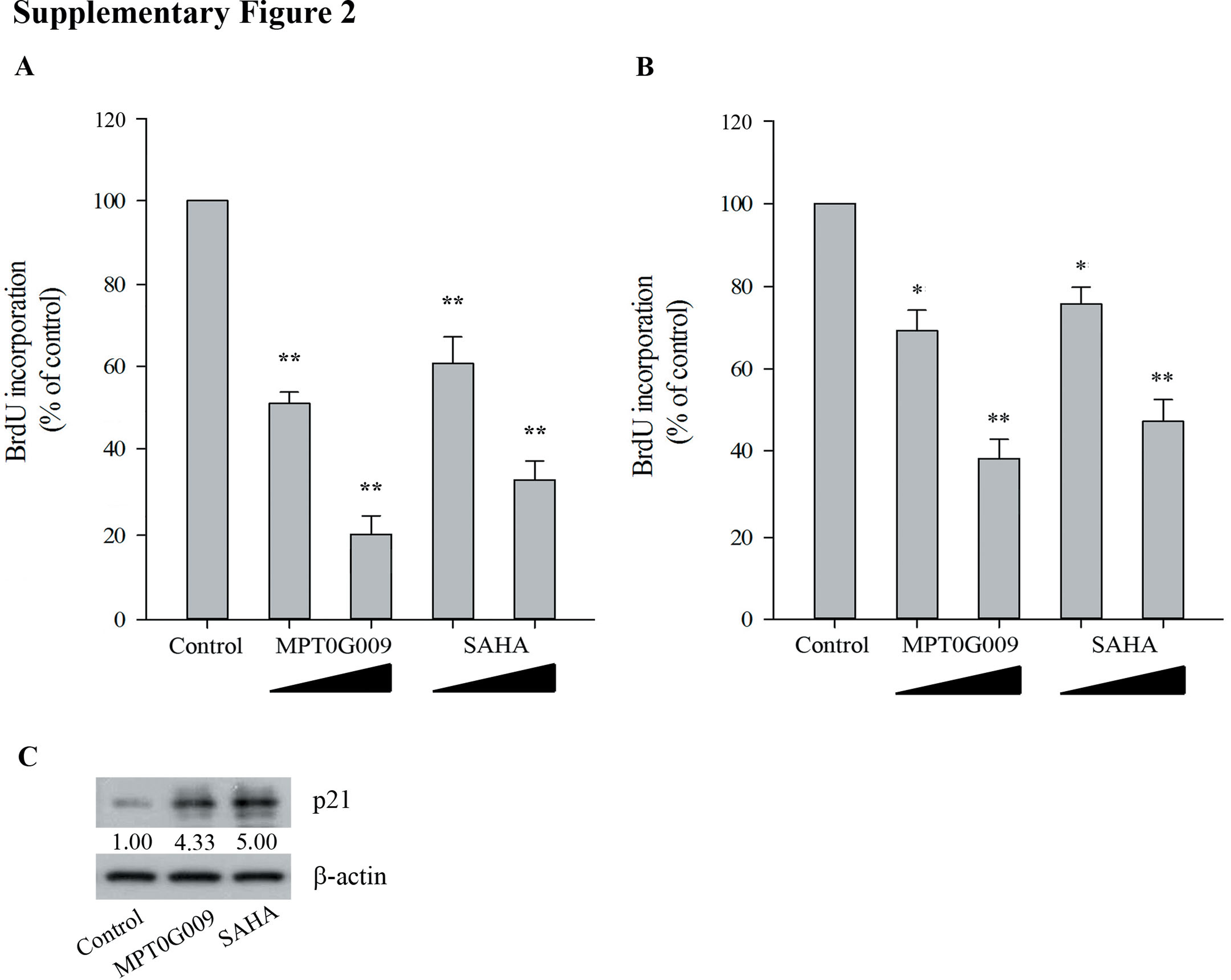

Supplement: Supplementary Figure 2 [file cddis2014133x2.tif]
