# Supplementary material for: Preclinical anti-arthritic study and pharmacokinetic properties of a potent histone deacetylase inhibitor MPT0G009
Source: Cell Death Dis. 2014 Apr 10;5(4):e1166–. doi: 10.1038/cddis.2014.133 (PMC5424110; doi:10.1038/cddis.2014.133)
Supplement: Supplementary Figure Legends [file cddis2014133x3.doc]

**Supplementary legends to figures**

**Supplementary Figure 1** MPT0G009 suppresses fibroblast-like synoviocytes proliferation and inflammation in the adjuvant-induced arthritis (AIA) model. Rats were orally treated with the vehicle, MPT0G009 (25 mg/kg), or suberoylanilide hydroxamic acid (SAHA; 200 mg/kg) after the onset of arthritis from day 2 to 21 (19 days). Subsequently, photomicrographs of ankle joint sections from the different groups by immunohistochemically staining with KI-67 (upper panel; images represent magnification at 100×) or COX-2 (lower panel; images represent magnification at 200×) antibodies. Scale bar = 100 and 50 μm respectively.

**Supplementary Figure 2** MPT0G009 inhibits the cell proliferation in HIG-82 synoviocytes and RA-FLS. (**a**) HIG-82 synoviocytes (2 × 10^4^) or (**b**) RA-FLS (2 × 10^4^) were treated with MPT0G009 (1 or 10 μM) or SAHA (3 or 30 μM) for 24 h, followed evaluation by BrdU assay. (**c**) RA-FLS (1 × 10^6^) were either untreated or were incubated for 24 h with MPT0G009 (1 μM) or SAHA (3 μM). Then cells were harvested and cell lysates were prepared for Western blot analysis of the indicated proteins. Results in (**a**, **b**) are the means ± SEM’s for three independent experiments. * *p* < 0.05 and ** *p* < 0.01 compared with control group. The numbers below each blot are the mean quantitative results as measured by densitometry relative to that without MPT0G009 or SAHA.
